# Low-dose cyclophosphamide combined with standard immunosuppressive therapy improves early response rates in severe aplastic anemia

**DOI:** 10.3389/fimmu.2026.1741042

**Published:** 2026-01-30

**Authors:** Hong Pan, Zhen Gao, Lele Zhang, Weiwang Li, Ruonan Li, Jingyu Zhao, Xiao Yu, Zhexiang Kuang, Neng Nie, Jianping Li, Yuan Li, Xingxin Li, Jinbo Huang, Xin Zhao, Jing Zhang, Meili Ge, Yizhou Zheng, Liwei Fang, Jun Shi

**Affiliations:** 1 State Key Laboratory of Experimental Hematology, National Clinical Research Center for Blood Diseases, Haihe Laboratory of Cell Ecosystem, Institute of Hematology & Blood Diseases Hospital, Chinese Academy of Medical Sciences & Peking Union Medical College, Tianjin, China; 2 Tianjin Institutes of Health Science, Tianjin, China; 3 Red Blood Cell Diseases Center & Regenerative Medicine Clinic, Institute of Hematology & Blood Diseases Hospital, Chinese Academy of Medical Sciences & Peking Union Medical College, Tianjin, China

**Keywords:** anti-thymocyte globulin, cyclophosphamide, immunosuppressive therapy, sever aplastic anemia, thrombopoietin receptor agonists

## Abstract

**Background:**

Thrombopoietin receptor agonists combined with anti-thymocyte globulin (ATG) and cyclosporine (CsA) are the standard immunosuppressive therapy (IST) for severe/very severe aplastic anemia (SAA/VSAA). However, early response rates remain suboptimal. Cyclophosphamide (CTX) has shown efficacy in relapsed/refractory AA. Therefore, we designed a clinical trial to evaluate low-dose CTX combined with the standard IST as a first-line treatment for SAA/VSAA to improve early response rates.

**Methods:**

This study was a single-arm, prospective, phase II clinical trial using a Simon’s two-stage design, and 43 patients were enrolled. The primary endpoint was the overall response rate (ORR) at 3 months. Newly diagnosed SAA/VSAA patients received a combination treatment as follows: porcine ATG at 25 mg/kg/day from days 1 to 5, CsA at 3–5 mg/kg/day continuously, hetrombopag at 15 mg/day starting from day 1 and continued for 6 months, low-dose CTX at 20 mg/kg/day on days 29–30 and days 43-44.

**Results:**

All 43 patients achieved the primary endpoint, demonstrating 3-month and 6-month ORR of 65.1% (28/43) and 69.8% (30/43) respectively. Complete response (CR) rates were 9.3% (4/43) at 3-month and 27.9% (12/43) at 6-month. CTX associated toxicities comprised 100% grade 1–2 gastrointestinal reactions, grade 3–4 neutropenia in 62.8% of patients (median duration 6 days, range 4-33). Infectious events occurred in 60.5% (26/43) of patients within the first 3 months of treatment, while no mortality observed during this period.

**Conclusions:**

Low-dose CTX combined with standard IST appears to improve the early response rate in SAA/VSAA patients with manageable toxicity.

## Introduction

Acquired aplastic anemia (AA) is a bone marrow failure disorder primarily mediated by immune dysregulation. Severe aplastic anemia, characterized by life-threatening cytopenia leading to hemorrhage, infections, and an extremely high one-year mortality rate if untreated (1), is managed with potent IST combining ATG and cyclosporine, which remains the standard non-transplant approach (2–4). The addition of Thrombopoietin receptor agonists (TPO-RAs) to this regimen has significantly improved both response rates and the quality of hematologic response, establishing it as the current frontline therapy (5, 6). A Phase III trial of horse ATG combined with cyclosporine and eltrombopag reported a 3-month overall response rate (ORR) of 59% in SAA (6). However, horse ATG is unavailable in China, and hepatic intolerance often limits the use of full-dose eltrombopag in Chinese patients. Consequently, another TPO-RA hetrombopag combined with porcine ATG and cyclosporine has emerged as a widely adopted IST regimen in China. Studies of this protocol report a 3-month response rate of 40–50% (7, 8), indicating that approximately half of patients remain transfusion-dependent at this critical timepoint. Thus, improving early efficacy in SAA remains an unmet clinical need to reduce transfusion dependency.

CTX has a potent cytotoxic effect on lymphocytes but minimal impact on hematopoietic stem cells. Due to its ability to target both B and T lymphocytes, CTX has been widely used in the treatment of various autoimmune diseases. Clinical studies have confirmed that high-dose cyclophosphamide ± cyclosporine achieves efficacy comparable to ATG in treating SAA, with a lower risk of relapse (9, 10). However, the toxic side effects of high-dose CTX limit its application in AA.

Based on the above rationale, we hypothesize that the addition of low-dose CTX to standard IST combined with hetrombopag may further enhance early response rates in patients with SAA.

## Methods

### Ethics and implementation

This investigator-initiated study was approved by the institutional ethics committee of Institute of Hematology & Blood Diseases Hospital, Chinese Academy of Medical Sciences & Peking Union Medical College (IIT2023038-EC-2) and registered on ClinicalTrials.gov (NCT05975996). All aspects of this study—including manuscript drafting, data acquisition, and statistical analysis—were exclusively performed by the authors. The data and its interpretation are fully validated, and protocol adherence was rigorously maintained throughout the research. Ethical compliance was ensured through written informed consent from every patient or their legal guardian. This research complied with the Declaration of Helsinki.

### Study design

This was a single-center, non-randomized, phase II clinical trial initiated by investigators. This study employed Simon’s two-stage optimal design to estimate the required sample size, with the 3-month ORR as the primary endpoint. The null hypothesis (H_0_) assumed a 3-month ORR of 45% for the standard IST and hetrombopag regimen, while the alternative hypothesis (H_1_) projected an ORR of 65%. Using R software (version 4.0.2) with a one-sided significance level (α) of 0.05 and a target power ≥80%, the two-stage design yielded a total sample size of 43 patients. Enrollment would be halted for futility if less than 9 responses were observed in the first 22 patients (stage 1).

### Patients

**Figure 1:**
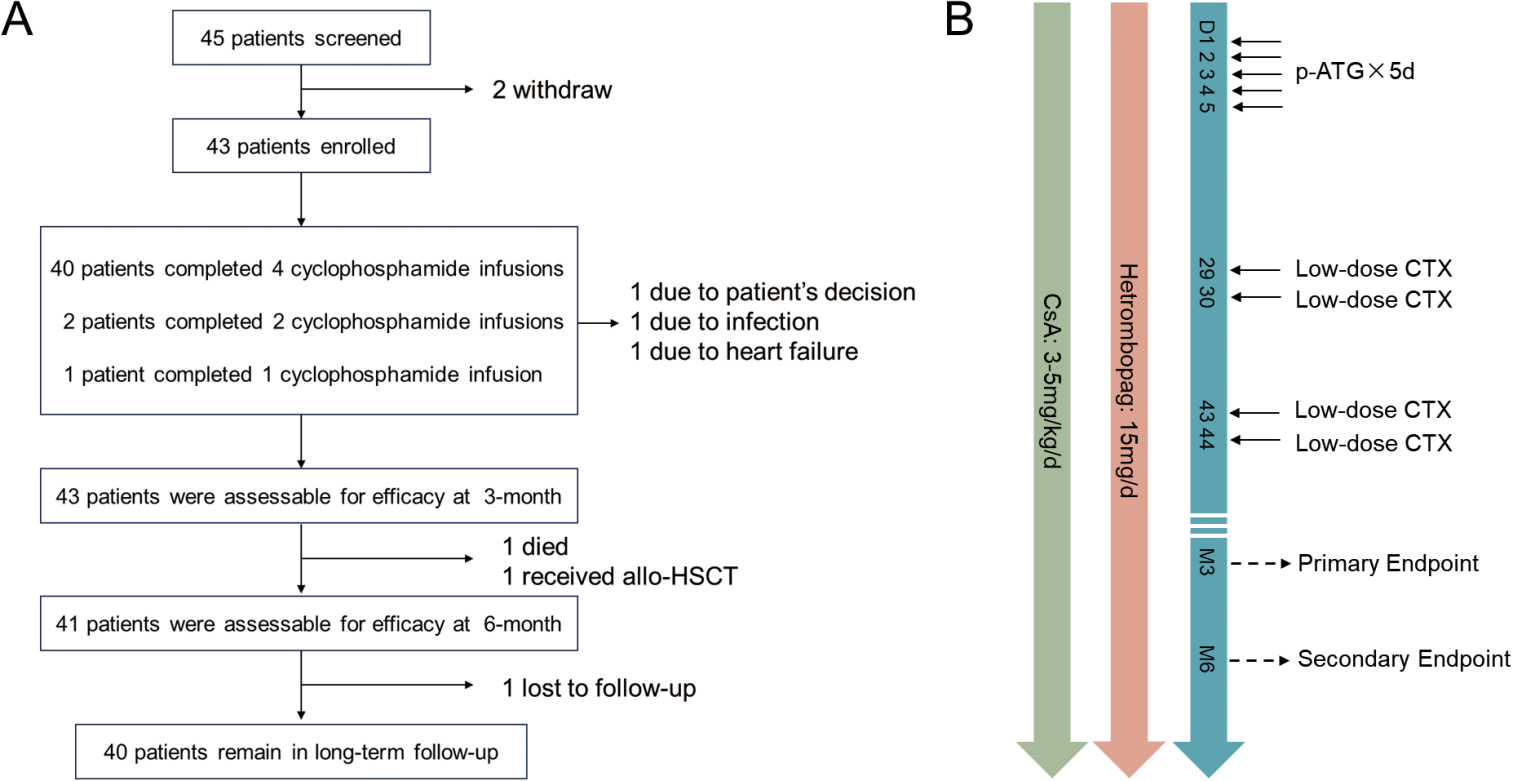
Trail profile. **(A)** Participant Flow Diagram: A total of 45 severe/very severe aplastic anemia (SAA/VSAA) patients were screened to enroll in this clinical trial. 2 patients withdrew consent for personal reasons before treatment. 3 patients failed to complete the full course of scheduled cyclophosphamide infusions. 1 patient with a 3-month PR response died accidentally in the fourth month. 1 VSAA patient proceeded to allogeneic hematopoietic stem cell transplantation (HSCT) due to refractory disease after 3 months of therapy. **(B)** Schematic Diagram: p-ATG was administered at 25 mg/kg/day. Cyclophosphamide (CTX) was administered at a dose of 20 mg/kg/day on days 29–30 and 43–44.

From July 2023 to September 2024, a total of 43 patients were enrolled in this study (**Figure 1A**). The inclusion criteria for this study were as follows: (1) patients aged ≥12 years; (2) confirmed diagnosis of treatment-naïve acquired severe or very severe aplastic anemia; (3) ineligibility or unwillingness to undergo allogeneic hematopoietic stem cell transplantation; (4) Eastern Cooperative Oncology Group (ECOG) performance status score ≤2; and (5) ability to fully understand the study protocol and voluntarily provide written informed consent. Patients were excluded from the study if they met any of the following criteria: (1) prior treatment with TPO-RA for >4 weeks before enrollment; (2) prior immunosuppressive therapy for >4 weeks before treatment initiation; (3) intolerance to cyclophosphamide or hetrombopag; (4) allergy to porcine ATG; (5) uncontrolled active infection; (6) uncontrolled hypertension (≥140/90 mmHg) or diabetes mellitus (fasting blood glucose ≥7.0 mmol/L or random blood glucose ≥11.1 mmol/L); (7) abnormal liver or kidney function, defined as aspartate aminotransferase (AST) or alanine aminotransferase (ALT) >3× the upper limit of normal (ULN), or serum creatinine ≥2.5×ULN; (8) history of chemotherapy/radiotherapy for malignant solid tumors; (9) history of other severe systemic diseases; (10) pregnancy, lactation, or women with childbearing potential; or (11) considered ineligible by investigators due to other factors that might affect study completion.

SAA was defined as bone marrow cellularity of less than 25% and decreased values for at least two of three blood counts (reticulocyte count <20×10^9^ cells/L, platelet count <20×10^9^ cells/L, and neutrophil count<0.5×10^9^ cells/L). Aplastic anemia was considered very severe if the patient met the criteria for SAA and had a neutrophil count less than 0.2 ×10^9^ cells/L. Congenital hematopoietic failure must be excluded. Patients under the age of 50 are required to undergo genetic screening for congenital bone marrow failure-related mutations as part of the diagnostic process. Patients aged 50 and above must test negative in both the mitomycin C (MMC) and comet assays to rule out potential congenital genetic factors before enrollment.

### Treatment regimen

Following enrollment, patients were administered a combined immunosuppressive regimen consisting of porcine ATG at 25 mg/kg/day on days 1–5, CsA 3–5 mg/kg/day maintained throughout the treatment course, and hetrombopag 15 mg/day initiated on day 1 and continued for 6 months. Sequential low-dose CTX (20 mg/kg/day) was subsequently administered on days 29–30 and 43–44 (**Figure 1B**). Supportive care, including infection prophylaxis and transfusion management, was provided in accordance with institutional guidelines. Infection prophylaxis and management include: for patients with an absolute neutrophil count (ANC) <0.5×10^9^/L, administer granulocyte colony-stimulating factor (G-CSF) at 2–5 μg/kg daily until ANC recovers to >5×10^9^/L, and initiate posaconazole for antifungal prophylaxis. If fever develops during treatment, prompt pathogen-directed testing and early empirical anti-infective therapy should be pursued based on clinical presentation.

### End points

The primary endpoint was 3-month overall response rate (ORR), defined as the proportion of patients achieving any of the following hematologic responses by 3 months after treatment initiation: complete response (CR), very good partial response (VGPR), good partial response (GPR), partial response (PR). The secondary endpoints comprised efficacy and safety outcomes. Efficacy endpoints included the 3-month CR rate and superior response rate (CR +VGPR+ GPR), as well as the 6-month ORR, CR rate, superior remission rate, and time to superior remission. Safety endpoints encompassed early all-cause mortality within 3 months, grade ≥3 treatment-related adverse events (per CTCAE v5.0), and clonal evolution (assessed via cytogenetic or molecular profiling).

Efficacy assessment criteria were derived from the Guidelines for the Diagnosis and Management of Adult Aplastic Anemia (1), refined further through our clinical experience. CR was defined as hemoglobin > 10 g/dL, absolute neutrophil count > 1.0×10^9/L, and platelet count > 100×10^9/L. VGPR was defined as hemoglobin > 10 g/dL, absolute neutrophil count > 1.0×10^9/L, and platelet count > 80×10^9/L. GPR was defined as hemoglobin > 8 g/dL, absolute neutrophil count > 1.0×10^9/L, and platelet count > 50×10^9/L. PR was defined as no longer meeting the criteria for SAA and transfusion independence but not meeting any of the response criteria defined above. Continuous transfusion dependency was classified as no response (NR). Superior remission includes CR, VGPR, and GPR, whereas inferior remission encompasses PR.

### Statistical analysis

Both primary and secondary endpoints were analyzed according to the intention-to-treat (ITT) principle, with the analysis population comprising all patients who received at least one dose of the investigational drug.

For descriptive statistical analyses, unless otherwise specified, categorical data will be presented as the number of subjects (n) and percentage (%) for each category level. For continuous data, the number of non-missing subjects (n), mean, standard deviation, median, minimum, and maximum will be reported as appropriate based on the specific context. The endpoints of rate were reported with 95% CIs, calculated with Clopper–Pearson binomial CIs. Time-to-event data were estimated using KaplanMeier analysis and presented with accompanying 95% CI.

## Results

### Patients

Among the 43 enrolled patients, 30.2% (13/43) were diagnosed with VSAA. The baseline characteristics of the cohort are detailed in **Table 1**. The median follow-up time was 14.9 months (range, 4.6–20.7 months). 45 patients were screened, with two patients withdrawing informed consent prior to treatment initiation. Among the 43 enrolled patients, 40 (93.0%) received four cyclophosphamide infusions per protocol, 2 (4.7%) received two infusions, and 1 (2.3%) received one infusion.

**Table 1:** Clinical characteristics at baseline.

Characteristics	All patients (n=43)	SAA patients (n=30)	VSAA patients (n=13)
Age — yr			
Median	33	34	22
Range	14-68	15-67	14-68
Sex — no. (%)			
Male	21(48.8)	15(50.0)	6(46.2)
Female	22(51.2)	15(50.0)	7(53.8)
Interval between onset and treatment — no. (%)			
<6 mo	39(90.7)	26(86.7)	13(100.0)
≥6 mo	4(9.3)	4(13.3)	0(0.0)
Laboratory values — Median (Range)			
RET count —×10^9^/L	18.3(2.4-46.0)	24.6(4.9-46.0)	6.5(2.4-19.0)
ANC count — ×10^9^/L	0.36(0.00-1.02)	0.40(0.20-1.02)	0.14(0.00-0.20)
PLT count — ×10^9^/L	8.0(1.0-32.0)	9.0(1.0-32.0)	5.5(2.0-11.0)
Ferritin — ng/mL	486.4(118.0-7228.0)	488.6(118.0-2648.0)	486.4(194.4-7228.0)
sTfR — mg/L	0.64(0.30-1.51)	0.66(0.45-1.51)	0.51(0.30-0.65)
With PNH clone — no. (%)	18(41.9)	14(46.7)	4(40)
Cytogenetic abnormalities — no. (%)			
Normal	40(93.0)	28(93.3)	12(92.3)
Abnormal karyotype	3(7.0)	2(6.7)	1(7.7)
With somatic mutation —no. (%)			
yes	9(20.9%)	7(23.3)	2(15.4)
no	34(79.1%)	23(76.7)	11(84.6)

SAA, severe aplastic anemia; VSAA, very severe aplastic anemia; RET, reticulocyte; ANC, neutrophil; PLT, plate; sTfR, soluble transferrin receptor PNH, paroxysmal nocturnal hemoglobinuria.

### Hematologic response

**Figure 2:**
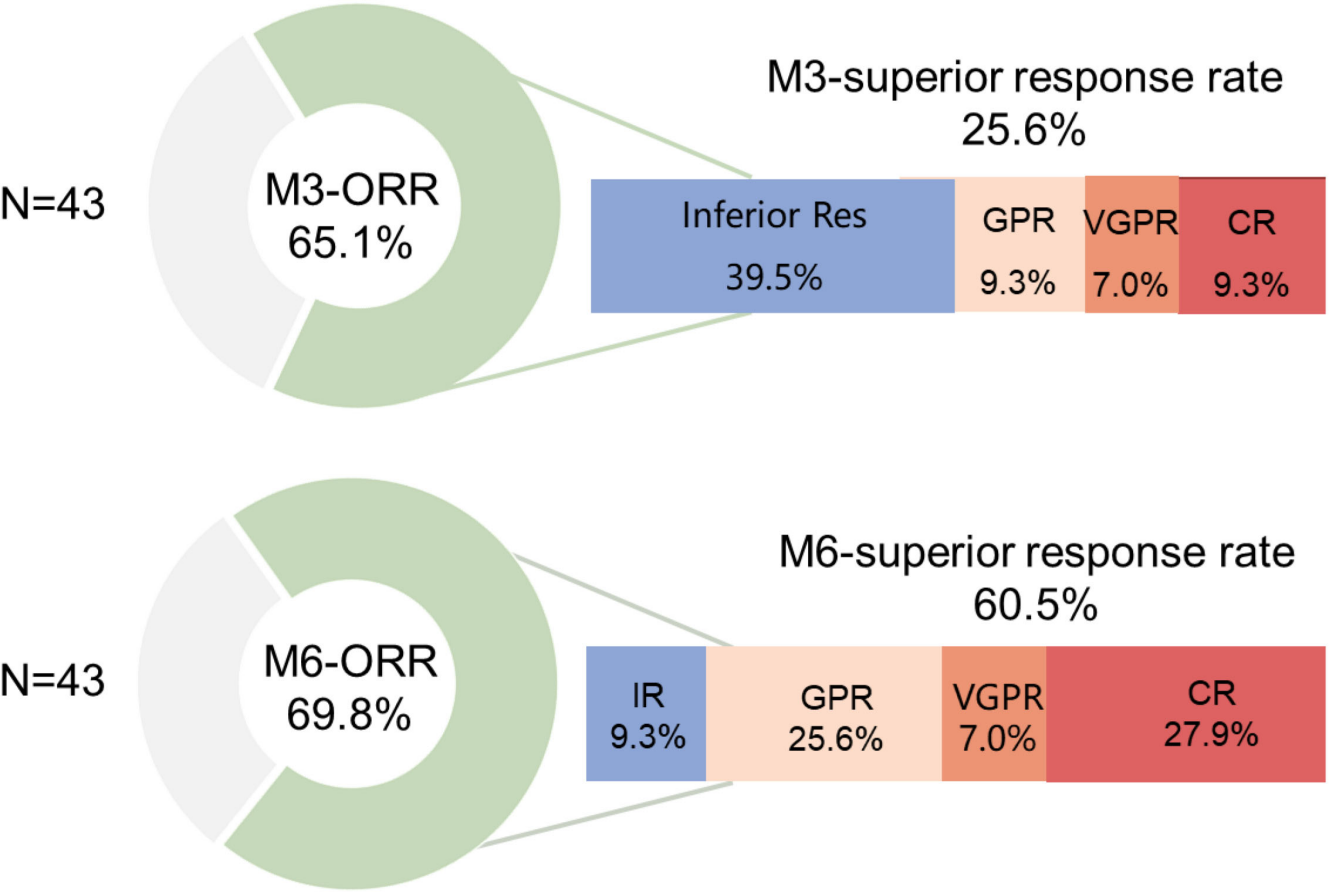
Overall hematological response rate at 3 months (primary endpoint) and 6 months (secondary endpoint). ORR: overall hematological response rate; IR: inferior response; GPR: good partial response; VGPR: very good partial response; CR: complete response. All 43 patients were assessable for 3-month efficacy, and 41 patients (including 1 due to accidental death and 1 who underwent (HSCT) were assessable for 6-month efficacy. Both time points were evaluated according to the intent-to-treat (ITT) principle.

The primary endpoint of this study, the 3-month ORR, was 65.1% (28/43, 95%CI=49.1-79.0). The 3-month superior remission rate and CR rate were 25.6% (11/43, 95%CI=13.5-41.2) and 9.3% (4/43, 95%CI=2.6-22.1), respectively. At the 6-month follow-up, the ORR showed no significant improvement compared to the 3-month assessment (69.8%, 30/43, 95%CI=53.9-82.8). However, the superior remission rate increased to 60.5% (26/43, 95%CI=44.4-75.1), with the CR rate rising to 27.9% (12/43, 95%CI=15.3-43.7) (**Figure 2**). The median time to first PR was 86 days (95% CI = 74–109), while the median time to first superior remission was 131 days (95% CI = 100–181).

**Figure 3:**
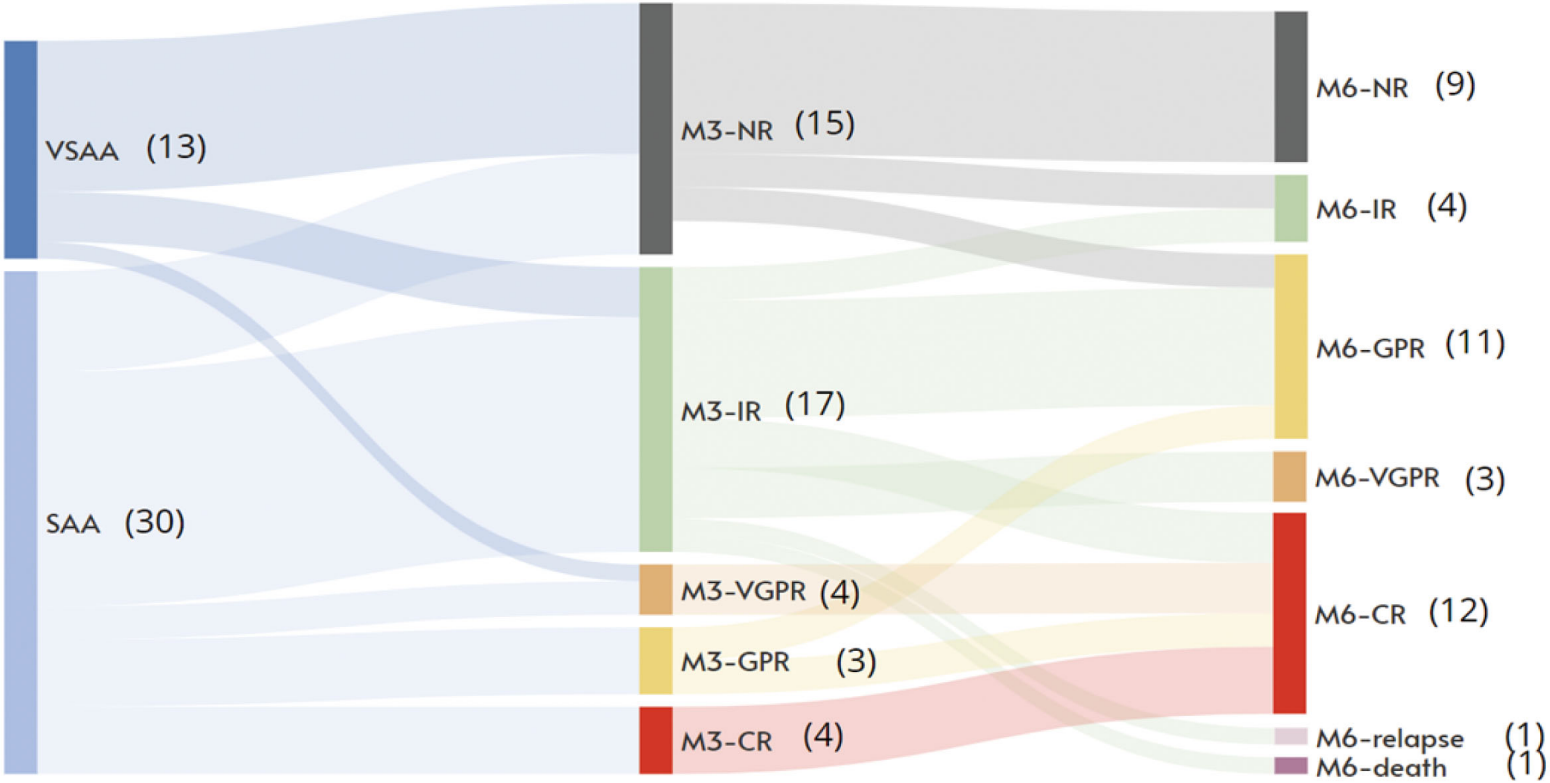
Kinetics of hematologic response. The Sankey diagram illustrates the responses of patients with SAA and VSAA at 3-month and 6-month. At the 3-month timepoint, 15 patients (9 with VSAA, 6 with SAA) remained in NR status. 1 patient opted for HSCT before reaching the 6-month evaluation, while among the remaining 14 NR patients, 4 achieved a response by 6 months. At 3 months, 11 patients were in a superior response state (GPR+VGPR+CR), and this number increased to 26 patients by the 6-month follow-up.

Compared with patients with SAA, those with VSAA demonstrated a significantly lower 3-month ORR (30.8% vs. 80.0%; *p* = 0.0041). From the hematologic response kinetics (**Figure 3**), it can be observed that among the 15 patients who showed no response at 3 months, 10 patients (66.7%) still failed to achieve hematologic response by 6 months. In contrast, among the 17 patients who achieved inferior remission at 3 months, 13 patients (76.5%) progressed to superior remission (including 3 CR), 2 patients (11.8%) remained in inferior remission, while 1 patient (5.9%) relapsed and 1 patient (5.9%) died due to an unrelated accident.

### Adverse events

The adverse events associated with hetrombopag, p-ATG, and cyclosporine in this study were consistent with standard immunosuppressive therapy (IST), primarily manifesting as serum sickness-like reactions and liver function abnormalities. With the addition of cyclophosphamide, investigator-assessed CTX-related adverse events included alopecia (Grade 1, 48.8%), nausea (Grades 1-2, 100%), neutropenia (Grades 1-4, Grade 3 or higher in 62.8%), and infections (Grades 1-3, Grade 3 in 25.6%), as detailed in **Table 2**. Neutrophil decline was observed in both CTX treatment cycles (Cycle 1: days 29-30; Cycle 2: days 43-44), with a median neutropenia duration of 6 days (range 0–30 days) in Cycle 1 and 4.5 days (range 0–28 days) in Cycle 2 (**Supplementary Figure S1A**). Three patients with VSAA experienced persistently low neutrophil counts. However, these patients were already in a state of granulocyte deficiency prior to CTX treatment and remained in a state of persistent neutropenia after therapy. No reduction in hemoglobin or platelets attributable to CTX was observed. The overall infection rate within 3 months of treatment was 60.5%, and 46% (20/43) of patients developed new infections post-CTX, predominantly febrile neutropenia (16%, 7/43), pneumonia (12%, 5/43), and upper respiratory tract infections (9%, 4/43) (**Supplementary Figure S1B**). No death was observed during the 3-month follow-up.

**Table 2:** Most common adverse events attributed to cyclophosphamide.

Events	Grade	Patients No (%)
Alopecia	1	21(48.8)
Nausea	1	36(83.7)
	2	7(16.3)
Neutropenia	1	7(16.3)
	2	4(9.3)
	3	6(14.0)
	4	21(48.8)
Infection	1	13(30.2)
	2	2(4.7)
	3	11(25.6)

#Infection: Refers to the occurrence of infections within 3 months from enrollment.

### Molecular and cytogenetic profiling and evolution

**Figure 4:**
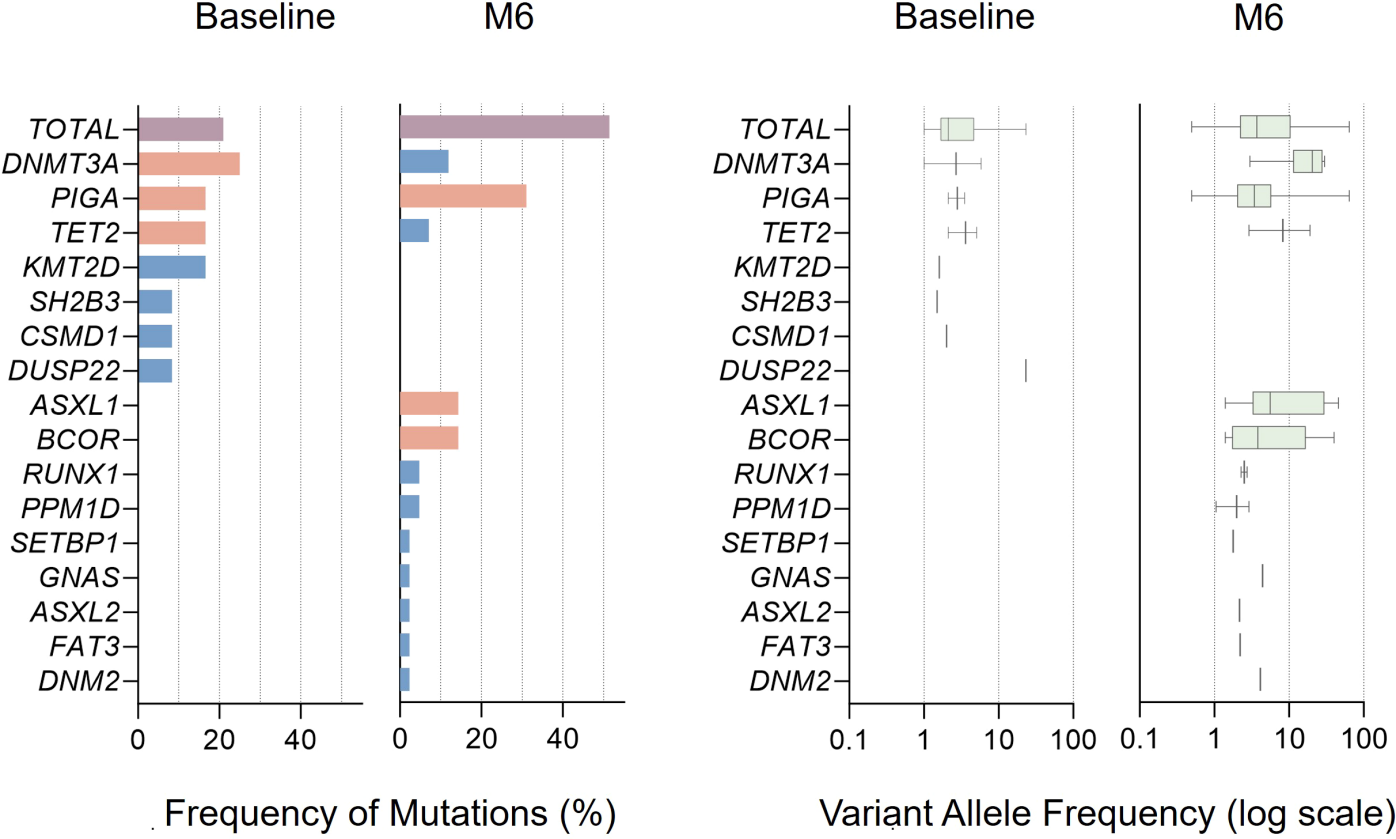
Somatic mutations profiling and evolution. This figure shows the frequency and variant allele frequency (VAF) of mutations at baseline, 6 months. The variant allele frequency of the mutations is shown on a logarithmic scale. The box-and-whisker plots of the specific gene mutations are shown; the whiskers indicate the range, the sides of the boxes indicate the interquartile range, and the vertical line within each box indicates the median.

In the cohort, 20.9% of patients harbored at least one Type I or II gene mutation prior to treatment (**Table 1**). The top three mutated genes were *DNMT3A* (25.0%), *PIGA* (16.7%), and *TET2* (16.7%); however, the variant allele frequency (VAF) of these genes across the entire cohort were each below 10% (**Figure 4**). By the 6-month time point, the proportion of patients harboring Type I/II somatic mutations increased to 51.4% (18/35), with the most frequently mutated genes being *PIGA* (31.0%), *ASXL1* (14.3%), and *BCOR* (14.3%). In addition to shifts in mutation spectrum, the variant allele frequencies (VAFs) of these mutations also rose compared to baseline levels (**Figure 4**). No progression to myelodysplastic syndromes or acute myeloid leukemia was observed in any patient as of the last follow-up date.

At baseline, 3 patients exhibited abnormal karyotypes: -Y, del (13) (q12q22), and t(X;19) (p10; p10). By the 6-month follow-up, the patient with -Y achieved normalization of karyotype, while the other two patients retained their original abnormal karyotypes. 18 of 43 patients (41.9%) tested positive for PNH clones at baseline. By 3 months, 16 of 40 patients (40.0%) remained PNH clone-positive, and at 6 months, the positivity rate was 16/38 (42.1%).

## Discussion

This phase II trial evaluated a modified IST protocol combining low-dose CTX with conventional IST regimens, and the primarily endpoint was the 3-month ORR. We observed a 3-month ORR of 65.1%, which is higher than the 50.6% ORR reported in a phase III clinical trial of hetrombopag combined with porcine ATG and CsA (7). However, the 6-month ORR showed no significant improvement compared to the 3-month results (69.8% vs. 65.1%), while the complete response (CR) rate (27.9% vs. 9.3%) and superior remission rate (60.5% vs. 25.6%) at 6 months were markedly increased compared to the 3-month timepoint. Notably, although the 3-month ORR in our study seemed to have an improvement, the 6-month ORR and CR rates remained comparable to those reported in other clinical studies of standard IST regimens (6, 7). This suggests that the intensified immunosuppressive therapy incorporating low-dose CTX may enable earlier onset of response in more patients, but does not enhance the quality of remission, highlighting the critical role of TPO-RAs in achieving higher remission quality (5–8, 11, 12). This may be related to CTX’s role as an immunosuppressant, which simultaneously targets both B and T lymphocytes. More intensive IST may correct immune-mediated hematopoietic failure earlier, thereby enhancing therapeutic efficacy. However, due to the cytotoxic properties of CTX, the quality of early response observed in this study was not optimal. The continued use of TPO-RA in later stages helped improve the quality of patient remission.

Brodsky et al. first pioneered the use of high-dose CTX in patients with SAA and observed CR in 7 out of 10 patients (10). Subsequently, the same team applied high-dose CTX as frontline therapy in treatment-naïve SAA patients and reported a 2-year transfusion-free survival rate of 73% among 19 evaluable cases. With extended 10-year follow-up, the overall survival (OS) rate for newly diagnosed SAA patients reached 88%, while the clonal evolution rate remained below 5% (9, 13). However, study by Scheinberg et al. revealed that intermediate-dose CTX in SAA patients was associated with substantial toxicities, including a mortality rate as high as 14% (3/22) (14). In contrast to previous studies utilizing CTX and CsA, our investigation represents the first clinical effort to combine CTX with both ATG and CsA, thereby intensifying immunosuppressive potency. To mitigate toxicity concerns, we reduced the CTX dose to 20 mg/kg/day for 4 doses (total cumulative dose 80 mg/kg) and split the administration into two cycles administered two weeks apart (days 29–30 and 43-44). Despite this dose optimization, transient neutropenia (median duration of 6 days per cycle) was still observed. However, neutrophil counts were restored with G-CSF support in the majority of patients, and no treatment-related mortality occurred within the 3-month follow-up period. Although the incidence of infections increased compared to standard IST regimens, these events remained manageable with aggressive antimicrobial therapy and standardized supportive care.

In this study, we observed an increased incidence of somatic mutations at 6 months compared to baseline (from 20.9% to 51.4%), primarily involving genes such as *DNMT3A*, *PIGA*, and *TET2*. Additionally, both the proportion of patients carrying PNH clones and the clone size showed a progressive increase. These findings align with results from the phase III clinical trial of eltrombopag combined with IST, which similarly reported a rise in somatic mutations at 6 months (6). The researchers hypothesized that hematopoietic recovery might involve oligoclonal expansion. In other words, transient shifts or selective expansion of certain progenitor subsets may occur during hematopoietic recovery, which do not necessarily represent true clonal evolution. Notably, no clonal evolution (e.g., progression to myelodysplastic syndromes or acute myeloid leukemia) was detected by the last follow-up in our cohort. However, whether this regimen increases the risk of clonal evolution requires extended follow-up to determine.

This prospective phase II trial showed that the addition of low-dose CTX to ATG plus CsA and hetrombopag was beneficial in patients with SAA. The addition of low-dose CTX induced an early response and tolerable toxicity. This study has limitations inherent to its single-arm design and modest sample size. Additionally, the short follow-up precludes definitive conclusions about long-term risks such as relapse or clonal evolution. Moving forward, extended long-term follow-up will be essential, and we are planning to conduct a rigorously designed phase 3 randomized controlled trial to definitively evaluate the efficacy of CTX in newly diagnosed patients with SAA.

## Data Availability

The raw data supporting the conclusions of this article will be made available by the authors, without undue reservation.

## Supplementary material

Supplementary Figure 1 Cyclophosphamide-induced neutropenia and associated infections. A. Line graph: Displays the neutrophil levels before two cycles of CTX treatment and the neutrophil nadir following CTX administration in each cycle. B. Pie chart (left): 46% (20/43) of patients developed new-onset infections following CTX therapy. Bar chart (right): Distribution of infection types and corresponding incidence rates.
