# Supplementary figures and images for: Low-dose cyclophosphamide combined with standard immunosuppressive therapy improves early response rates in severe aplastic anemia
Source: Front Immunol. 2026 Jan 30;17:1741042. doi: 10.3389/fimmu.2026.1741042 (PMC12902132; doi:10.3389/fimmu.2026.1741042)

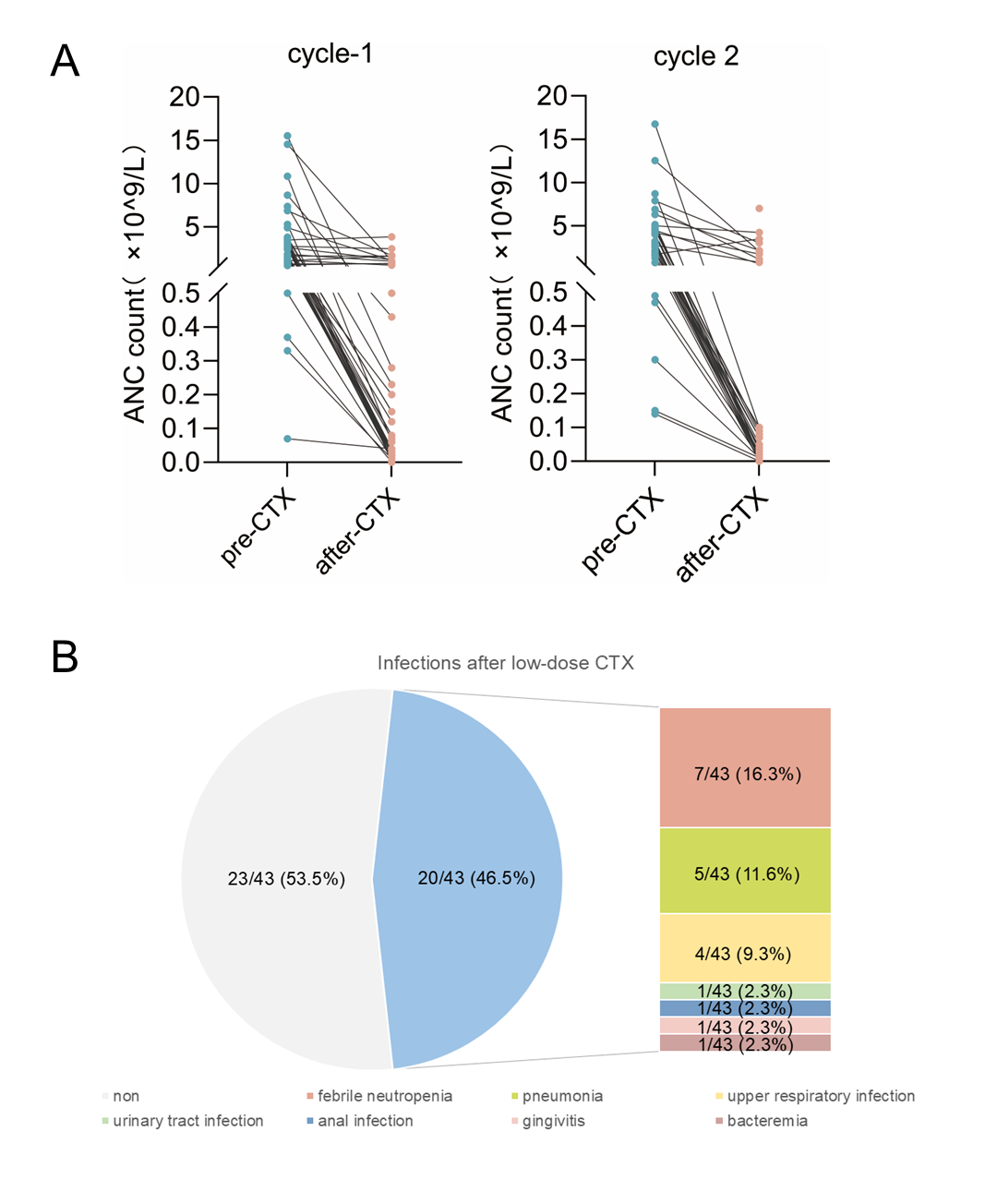

Supplement: Supplementary Figure 1 — Cyclophosphamide-induced neutropenia and associated infections. A. Line graph: Displays the neutrophil levels before two cycles of CTX treatment and the neutrophil nadir following CTX administration in each cycle. B. Pie chart (left): 46% (20/43) of patients developed new-onset infections following CTX therapy. Bar chart (right): Distribution of infection types and corresponding incidence rates. [file Image1.tiff]
